# Supplementary material for: The global distribution and environmental drivers of the soil antibiotic resistome
Source: Microbiome. 2022 Dec 12;10:219. doi: 10.1186/s40168-022-01405-w (PMC9743735; doi:10.1186/s40168-022-01405-w)
Supplement: Supplementary file 2 — Additional file 1: Supplementary Figure 1. Extrapolation of uncertainties associated with the global survey used in this study. Supplementary Figure 2. Main structure for the a priori structural equation model used in this study. Supplementary Figure 3. Structural equation models assessing the direct and indirect effects of environmental factors on the proportion (A) and richness (B) of ARGs in natural ecosystems only (i.e. croplands excluded, n = 802). Supplementary Figure 4. Global precipitation (A) and temperature (B) seasonality maps used in our study (https://www.worldclim.org/data/index.html). Supplementary Table 1. List of antibiotic resistance genes (ARGs) mobile genetic elements (MGEs) considered in this study. Supplementary Table 2. Biomes included in this study. The biome classification was done based on vegetation field information and climatic information from the Köppen classification16. Supplementary Table 3. Environmental factors included in our structural equation model. Supplementary Table 4. Standardized direct effects of SEM on the proportion of soil ARGs. Supplementary Table 5. Standardized direct effects of SEM on the richness of soil ARGs. [file 40168_2022_1405_MOESM1_ESM.pdf]

Supplementary Information for

The global distribution and environmental drivers of the soil antibiotic resistome

Manuel Delgado-Baquerizo, Hang-Wei Hu, Fernando T. Maestre, Carlos A. Guerra, Nico Eisenhauer, David J. Eldridge, Yong-Guan Zhu, Qing-Lin Chen, Pankaj Trivedi, Shuai Du, Thulani P. Makhalanyane, Jay P. Verma, Beatriz Gozalo, Victoria Ochoa, Sergio Asensio, Ling Wang, Eli Zaady, Javier G. Illán, Christina Siebe, Tine Grebenc, Xiaobing Zhou, Yu-Rong Liu, Adebola R. Bamigboye, José L. Blanco-Pastor, Jorge Duran, Alexandra Rodríguez, Steven Mamet, Fernando Alfaro, Sebastian Abade, Alberto L. Teixido, Gabriel F. Peñaloza-Bojacá, Marco A. Molina-Montenegro, Cristian Torres-Díaz, Cecilia Perez, Antonio Gallardo, Laura García-Velázquez, Patrick E. Hayes, Sigrid Neuhauser, Ji-Zheng He

***Corresponding authors:**
Manuel Delgado-Baquerizo. M.delgado.baquerizo@csic.es
Hang-Wei Hu. hang-wei.hu@unimelb.edu.au
Ji-Zheng He. jizheng.he@unimelb.edu.au

This PDF file includes:
Supplementary Figures 1-4
Supplementary Tables 1-5

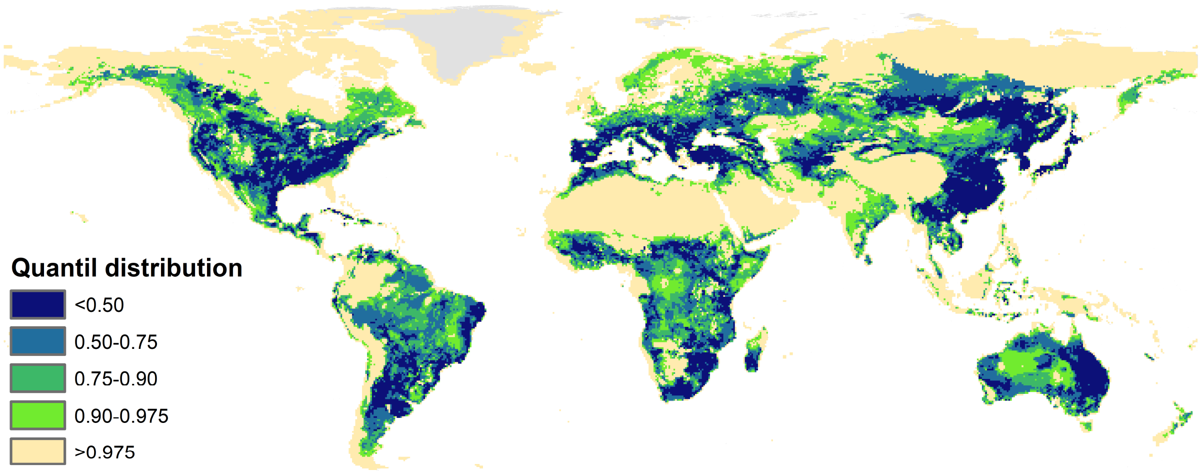

Supplementary Figure 1. Extrapolation of uncertainties associated with the global survey used in this study. Our map used the spatial accuracy indicator regarding the quantile distribution of the Chi-squared distribution with ten degrees of freedom based on the Mahalanobis multidimensional distance (corresponding to vegetation cover, annual precipitation, precipitation seasonality, mean annual temperature, temperature seasonality, soil texture, elevation, pH, soil carbon, and C:N ratio). This analysis provides information on those environmental conditions and locations of the planets which are underrepresented in our survey. Our dataset covers a wide range of the terrestrial environmental conditions found on Earth, being the rest considered as outliers (for this purpose an outlier is a location that has a value above the 97.5% quantile of the Chi-squared distribution).

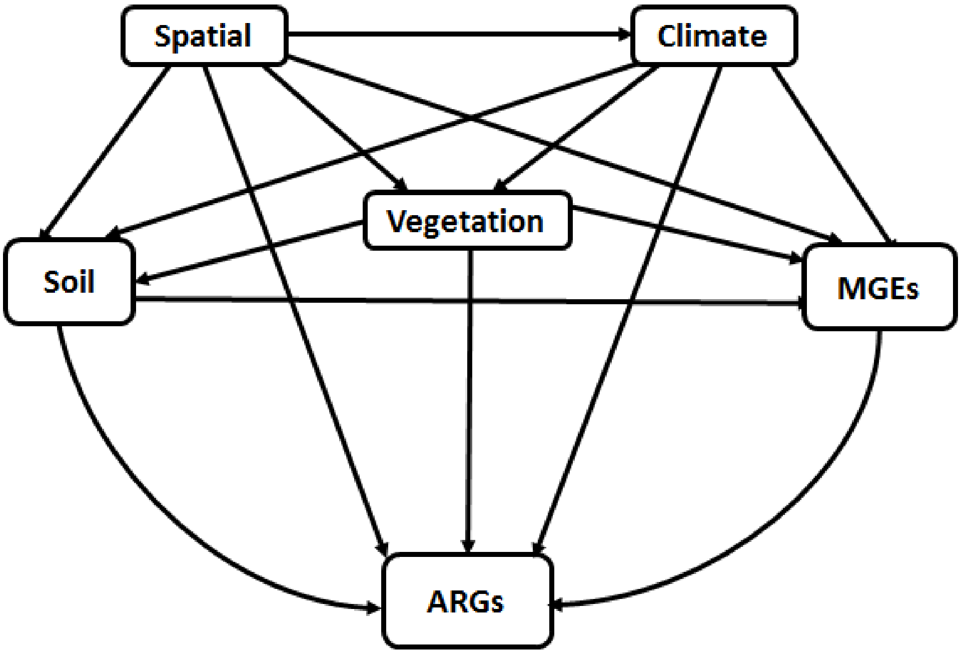

Supplementary Figure 2. Main structure for the *a priori* structural equation model used in this study. We grouped the different categories of predictors (space, climate, soil properties, vegetation and MGEs) in the same box for graphical simplicity (these boxes do not represent latent variables). See all considered direct associations in Supplementary Tables 4-5. Variables within these boxes are allowed to covary, with the exception of elevation and spatial dissimilarity, which constituted our degree of freedom.

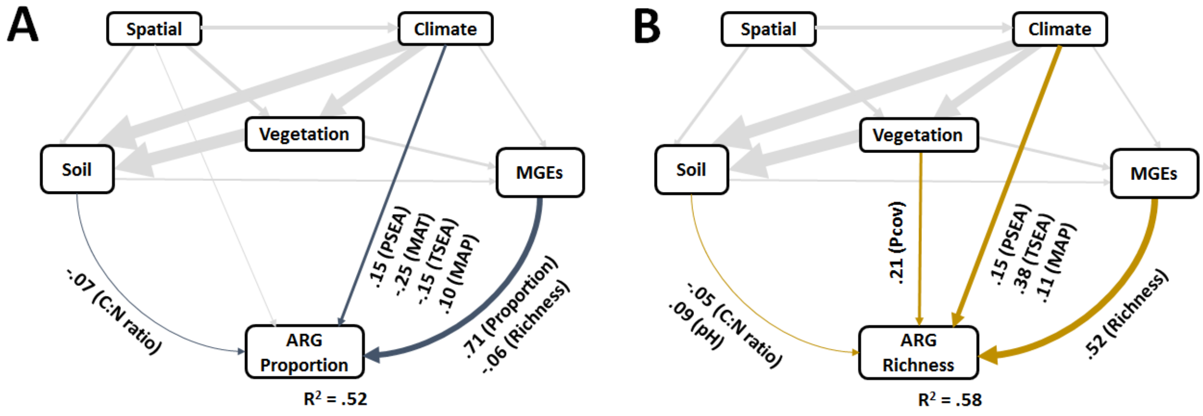

Supplementary Figure 3. Structural equation models assessing the direct and indirect effects of environmental factors on the proportion (A) and richness (B) of ARGs in natural ecosystems only (i.e. croplands excluded, n = 802). The proportion of ARGs was determined as the average standardized relative abundance of 285 individual ARGs. We grouped the different categories of predictors (climate, soil properties, vegetation and MGEs) in the same box for graphical simplicity (these boxes do not represent latent variables). Variables within these boxes are allowed to covary, with the exception of elevation and spatial dissimilarity, which constituted our degree of freedom. Numbers adjacent to arrows are indicative of the effect size of the relationship. Only significant paths ($P < 0.05$) are plotted. MGEs includes both richness and proportions.

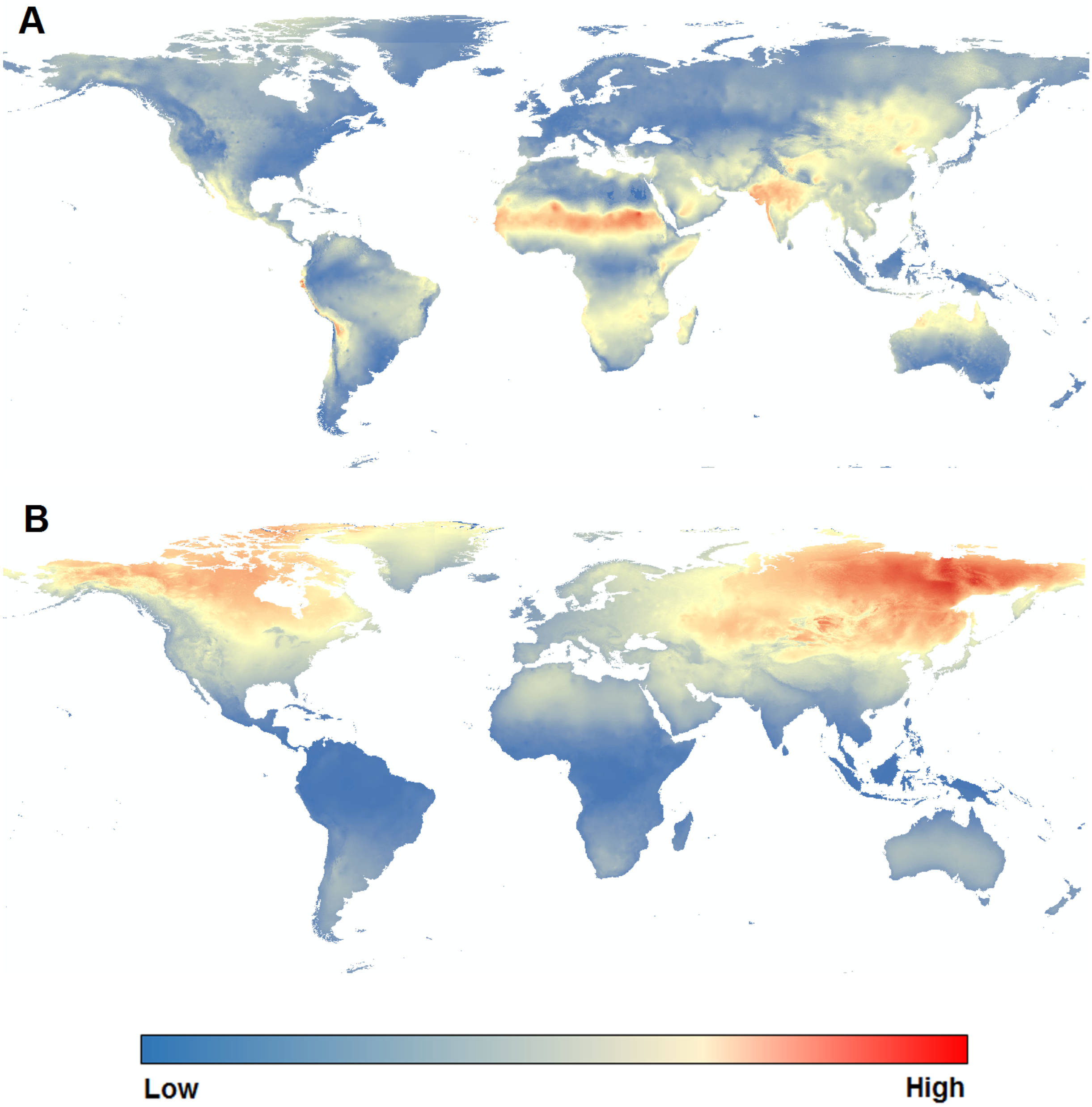

Supplementary Figure 4. Global precipitation (A) and temperature (B) seasonality maps used in our study (https://www.worldclim.org/data/index.html).

Supplementary Table 1. List of antibiotic resistance genes (ARGs) mobile genetic elements (MGEs) considered in this study.

Gene Name	Type	Gene Classification	Resistance Mechanism	Forward Primer	Reverse Primer
16S rRNA	16S rRNA	16S rRNA	Internal control	GGGTTGCGCTCGTTGC	ATGGYTGTCGTCAGCTCGTG
intI	MGES	MGE	integrase	GGCATCCAAGCAGCAAG	AAGCAGACTTGACCTGA
intI1	MGES	MGE	integrase	CGAACGAGTGGCGGAGGGTG	TACCCGAGAGCTTGGCACCCA
tnpA-03	MGES	MGE	transposase	AATTGATGCGGACGGCTTAA	TCACCAAACTGTTTATGGAGTCGTT
IS613	MGES	MGE	transposase	AGGTTCGGACTCAATGCAACA	TTCAGCACATACCGCCTTGAT
tnpA-01	MGES	MGE	transposase	CATCATCGGACGGACAGAATT	GTCGGAGATGTGGGTGTAGAAAGT
tnpA-04	MGES	MGE	transposase	CCGATCACGGAAAGCTCAAG	GGCTCGCATGACTTCGAATC
tnpA-07	MGES	MGE	transposase	GAAACCGATGCTACAATATCCAATTT	CAGCACCGTTTGCAGTGTAAG
tnpA-05	MGES	MGE	transposase	GCCGCACTGTCGATTTTTATC	GCGGGATCTGCCACTTCTT
Tp614	MGES	MGE	transposase	GGAAATCAACGGCATCCAGTT	CATCCATGCGCTTTTGTCTCT
tnpA-02	MGES	MGE	transposase	GGGCGGGTCGATTGAAA	GTGGGCGGGATCTGCTT
catB3	ARG	FCA	deactivate	GCACTCGATGCCTTCCAAAA	AGAGCCGATCCAAACGTCAT
catB8	ARG	FCA	deactivate	CACTCGACGCCTTCCAAAG	CCGAGCCTATCCAGACATCATT
cfr	ARG	FCA	deactivate	GCAAAATTCAGAGCAAGTTACGAA	AAAATGACTCCCAACCTGCTTTAT
floR	ARG	FCA	efflux	ATTGTCTTCACGGTGTCCGTTA	CCGCGATGTCGTCGAACT
cmlA1-01	ARG	FCA	efflux	TAGGAAGCATCGGAACGTTGAT	CAGACCGAGCACGACTGTTG
cmlA1-02	ARG	FCA	efflux	AGGAAGCATCGGAACGTTGA	ACAGACCGAGCACGACTGTTG
cmx(A)	ARG	FCA	efflux	GCGATCGCCATCCTCTGT	TCGACACGGAGCCTTGGT
catA1	ARG	FCA	deactivate	GGGTGAGTTTCACCAGTTTTGATT	CACCTTGTCGCCTTGCGTATA
qnrA	ARG	FCA	unknown	AGGATTTCTCACGCCAGGATT	CCGCTTTCAATGAAACTGCAA
aac	ARG	Aminoglycoside	deactivate	CCCTGCGTTGTGGCTATGT	TTGGCCACGCCAATCC
aacC1	ARG	Aminoglycoside	deactivate	GGTCGTGAGTTCGGAGACGTA	GCAAGTTCCCGAGGTAATCG
aacC2	ARG	Aminoglycoside	deactivate	ACGGCATTCTCGATTGCTTT	CCGAGCTTCACGTAAGCATTT
aacC4	ARG	Aminoglycoside	deactivate	CGGCGTGGGACACGAT	AGGGAACCTTTGCCATCAACT
aac(6')I1	ARG	Aminoglycoside	deactivate	GACCGGATTAAGGCCGATG	CTTGCCTTGATATTCAGTTTTTATAACCA
aacA/aphD	ARG	Aminoglycoside	deactivate	AGAGCCTTGGGAAGATGAAGTTT	TTGATCCATACCATAGACTATCTCATCA
aac(6')-Iy	ARG	Aminoglycoside	deactivate	GCTTTGCGGATGCCTCAAT	GGAGAACAAAAATACCTTCAAGGAAA
aac(6')-II	ARG	Aminoglycoside	deactivate	CGACCCGACTCCGAACAA	GCACGAATCCTGCCTTCTCA
aacC	ARG	Aminoglycoside	deactivate	CGTCACTTATTCGATGCCCTTAC	GTCGGGCGCGGCATA
aac(6')-Ib-01	ARG	Aminoglycoside	deactivate	GTTTGAGAGGCAAGGTACCGTAA	GAATGCCTGGCGTGTTTGA
aac(6')-Ib-02	ARG	Aminoglycoside	deactivate	CGTCGCCGAGCAACTTG	CGGTACCTTGCCTCTCAAACC
aadA5-01	ARG	Aminoglycoside	deactivate	ATCACGATCTTGCGATTTTGCT	CTGCGGATGGGCCTAGAAG
aadA5-02	ARG	Aminoglycoside	deactivate	GTTCTTGCTCTTGCTCGCATT	GATGCTCGGCAGGCAAAC
aac(6')-Ib-03	ARG	Aminoglycoside	deactivate	AGAAGCACGCCCGACACTT	GCTCTCCATTCAGCATTGCA

aadA1	ARG	Aminoglycoside	deactivate	AGCTAAGCGCGAACTGCAAT	TGGCTCGAAGATACCTGCAA
aadA2-01	ARG	Aminoglycoside	deactivate	ACGGCTCCGCAGTGGAT	GGCCACAGTAACCAACAAATCA
aadA-01	ARG	Aminoglycoside	deactivate	GTTGTGCACGACGACATCATT	GGCTCGAAGATACCTGCAAGAA
aadA2-02	ARG	Aminoglycoside	deactivate	CTTGTCGTGCATGACGACATC	TCGAAGATACCCGCAAGAATG
aadD	ARG	Aminoglycoside	deactivate	CCGACAACATTTCTACCATCCTT	ACCGAAGCGCTCGTCGTATA
aadA2-03	ARG	Aminoglycoside	deactivate	CAATGACATTCTTGCGGGTATC	GACCTACCAAGGCAACGCTATG
aadA9-01	ARG	Aminoglycoside	deactivate	CGCGGCAAGCCTATCTTG	CAAATCAGCGACCGCAGACT
aadA-02	ARG	Aminoglycoside	deactivate	CGAGATTCTCCGCGCTGTA	GCTGCCATTCTCCAAATTGC
aadA9-02	ARG	Aminoglycoside	deactivate	GGATGCACGCTTGGATGAA	CCTCTAGCGGCCGGAGTATT
aadE	ARG	Aminoglycoside	deactivate	TACCTTATTGCCCTTGGAAGAGTTA	GGAACTATGTCCCTTTTAATTCTACAATCT
spcN-01	ARG	Aminoglycoside	deactivate	AAAAGTTCGATGAAACACGCCTAT	TCCAGTGGTAGTCCCCGAATC
spcN-02	ARG	Aminoglycoside	deactivate	CAGAATCTTCCTGAAAAGTTTGATGAA	CGCAGACACGCCGAATC
aadA-1-01	ARG	Aminoglycoside	deactivate	AAAAGCCCGAAGAGGAACTTG	CATCTTTCACAAAGATGTTGCTGTCT
aph6ia	ARG	Aminoglycoside	deactivate	CCCATCCCATGTGTAAGGAAA	GCCACCGCTTCTGCTGTAC
aph(2')-Id-02	ARG	Aminoglycoside	deactivate	TGAGCAGTATCATAAGTTGAGTGAAAAG	GACAGAACAATCAATCTCTATGGAATG
aph(2')-Id-01	ARG	Aminoglycoside	deactivate	TAAGGATATACCGACAGTTTTGGAAA	TTTAATCCCTCTTCATACCAATCCATA
aph	ARG	Aminoglycoside	deactivate	TTTCAGCAAGTGGATCATGTTAAAAT	CCAAGCTGTTTCCACTGTTTTTC
aphA1	ARG	Aminoglycoside	deactivate	TGAACAAGTCTGGAAAGAAATGCA	CCTATTAATTTCCCCTCGTCAAAAA
aadA-1-02	ARG	Aminoglycoside	deactivate	CGGAATTGAAAAAACTGATCGAA	ATACCGGCTGTCCGTCATTT
str	ARG	Aminoglycoside	deactivate	AATGAGTTTTGGAGTGTCTCAACGTA	AATCAAAACCCCTATTAAAGCCAAT
strA	ARG	Aminoglycoside	deactivate	CCGGTGGCATTTGAGAAAAA	GTGGCTCAACCTGCGAAAAG
strB	ARG	Aminoglycoside	deactivate	GCTCGGTCGTGAGAACAATCT	CAATTTCGGTCGCCTGGTAGT
ampC-01	ARG	Beta Lactamase	deactivate	TGGCGTATCGGGTCAATGT	CTCCACGGGCCAGTTGAG
ampC-02	ARG	Beta Lactamase	deactivate	GCAGCACGCCCCGTAA	TGTACCCATGATGCGCGTACT
ampC-04	ARG	Beta Lactamase	deactivate	TCCGGTGACGCGACAGA	CAGCACGCCGGTGAAAGT
ampC-05	ARG	Beta Lactamase	deactivate	CTGTTCGAGCTGGGTTCTATAAGTAAA	CAGTATCTGGTCACCGGATCGT
ampC-06	ARG	Beta Lactamase	deactivate	CCGCTCAAGCTGGACCATAC	CCATATCCTGCACGTTGGTTT
ampC-07	ARG	Beta Lactamase	deactivate	CCGCCCAGAGCAAGGACTA	GCTCGACTTCACGCCGTAAG
ampC-09	ARG	Beta Lactamase	deactivate	CAGCCGCTGATGAAAAAATATG	CAGCGAGCCCACTTCGA
ampC/blaDHA	ARG	Beta Lactamase	deactivate	TGGCCGCAGCAGAAAGA	CCGTTTTATGCACCCAGGAA
bla1	ARG	Beta Lactamase	deactivate	GCAAGTTGAAGCGAAAGAAAAGA	TACCAGTATCAATCGCATATACACCTAA
bla-ACC-1	ARG	Beta Lactamase	deactivate	CACACAGCTGATGGCTTATCTAAAA	AATAAACGCGATGGGTTCCA
blaCMY	ARG	Beta Lactamase	deactivate	CCGCGGCGAAATTAAGC	GCCACTGTTTGCCTGTCAGTT
blaCMY2-01	ARG	Beta Lactamase	deactivate	AAAGCCTCAT GGGTGCATAAA	ATAGCTTTTGTTTGCCAGCATCA
blaCMY2-02	ARG	Beta Lactamase	deactivate	GCGAGCAGCCTGAAGCA	CGGATGGGCTTGTCCTCTT
blaCTX-M-01	ARG	Beta Lactamase	deactivate	GGAGGCGTGACGGCTTTT	TTCAGTGCGATCCAGACGAA
blaCTX-M-02	ARG	Beta Lactamase	deactivate	GCCGCGGTGCTGAAGA	ATCGGATTATAGTTAACCAGGTCAGATTT

blaCTX-M-03	ARG	Beta Lactamase	deactivate	CGATACCACCACGCCGTTA	GCATTGCCCAACGTCAGATT
blaCTX-M-04	ARG	Beta Lactamase	deactivate	CTTGGCGTTGCGCTGAT	CGTTCATCGGCACGGTAGA
blaCTX-M-05	ARG	Beta Lactamase	deactivate	GCGATAACGTGGCGATGAAT	GTCGAGACGGAACGTTTCGT
blaCTX-M-06	ARG	Beta Lactamase	deactivate	CACAGTTGGTGACGTGGCTTAA	CTCCGCTGCCGGTTTTATC
blaGES	ARG	Beta Lactamase	deactivate	GCAATGTGCTCAACGTTCAAG	GTGCCTGAGTCAATTCTTTCAAAG
blaIMP-01	ARG	Beta Lactamase	deactivate	AACACGGTTTGGTGGTTCTTGTA	GCGCTCCACAAACCAATTG
blaIMP-02	ARG	Beta Lactamase	deactivate	AAGGCAGCATTTCCTCTCATTTT	GGATAGATCGAGAATTAAGCCACTCT
bla-L1	ARG	Beta Lactamase	deactivate	CACCGGGTTACCAGCTGAAG	GCGAAGCTGCGCTTGTAGTC
blaMOX/blaCMY	ARG	Beta Lactamase	deactivate	CTATGTCAATGTGCCGAAGCA	GGCTTGTCCTCTTTCGAATAGC
blaOCH	ARG	Beta Lactamase	deactivate	GGCGACTTGCGCCGTAT	TTTTCTGCTCGGCCATGAG
blaOKP	ARG	Beta Lactamase	deactivate	GCCGCCATCACCATGAG	GGTGACGTTGTCACCGATCTG
blaOXA1/blaOXA30	ARG	Beta Lactamase	deactivate	CGGATGGTTTGAAGGGTTTATTAT	TCTTGGCTTTTATGCTTGATGTTAA
blaOXA10-01	ARG	Beta Lactamase	deactivate	CGCAATTATCGGCCTAGAAACT	TTGGCTTTCCGTCCCATTT
blaOXA10-02	ARG	Beta Lactamase	deactivate	CGCAATTATCGGCCTAGAAACT	TTGGCTTTCCGTCCCATTT
blaOXY	ARG	Beta Lactamase	deactivate	CGTTCAGGCGGCAGGTT	GCCGCGATATAAGATTTGAGAATT
blaPAO	ARG	Beta Lactamase	deactivate	CGCCGTACAACCGGTGAT	GAAGTAATGCGGTTCTCCTTTCA
blaPER	ARG	Beta Lactamase	deactivate	TGCTGGTTGCTGTTTTTGTGA	CCTGCGCAATGATAGCTTCAT
blaPSE	ARG	Beta Lactamase	deactivate	TTGTGACCTATTCCCCTGTAATAGAA	TGCGAAGCACGCATCATC
blaROB	ARG	Beta Lactamase	deactivate	GCAAAGGCATGACGATTGC	CGCGCTGTTGTCGCTAAA
blaSFO	ARG	Beta Lactamase	deactivate	CCGCCGCCATCCAGTA	GGGCCGCCAAGATGCT
blaSHV-01	ARG	Beta Lactamase	deactivate	TCCCATGATGAGCACCTTTAAA	TTCGTCACCGGCATCCA
blaSHV-02	ARG	Beta Lactamase	deactivate	CTTTCCCATGATGAGCACCTTT	TCCTGCTGGCGATAGTGGAT
blaTEM	ARG	Beta Lactamase	deactivate	AGCATCTTACGGATGGCATGA	TCCTCCGATCGTTGTCAGAAGT
blaTLA	ARG	Beta Lactamase	deactivate	ACACTTTGCCATTGCTGTTTATGT	TGCAAATTTCGGCAATAATCTTT
blaVEB	ARG	Beta Lactamase	deactivate	CCCGATGCAAAGCGTTATG	GAAAGATTCCCTTTATCTATCTCAGACAA
blaVIM	ARG	Beta Lactamase	deactivate	GCACTTCTCGCGGAGATTG	CGACGGTGATGCGTACGTT
blaZ	ARG	Beta Lactamase	deactivate	GGAGATAAAGTAACAAATCCAGTTAGATATGA	TGCTTAATTTTCCATTTGCGATAAG
cepA	ARG	Beta Lactamase	deactivate	AGTTGCGCAGAACAGTCCTCTT	TCGTATCTTGCCCGTCGATAAT
cfiA	ARG	Beta Lactamase	deactivate	GCAGCGTTGCTGGACACA	GTTCGGGATAAACGTGGTGACT
cfxA	ARG	Beta Lactamase	deactivate	TCATTCCTCGTTCAAGTTTTCAGA	TGCAGCACCAAGAGGAGATGT
cphA-01	ARG	Beta Lactamase	deactivate	GCGAGCTGCACAAGCTGAT	CGGCCCAGTCGCTCTTC
cphA-02	ARG	Beta Lactamase	deactivate	GTGCTGATGGCGAGTTTCTG	GGTGTGGTAGTTGGTGTTGATCAC
fox5	ARG	Beta Lactamase	deactivate	GGTTTGCCGCTGCAGTTC	GCGGCCAGGTGACCAA
mecA	ARG	Beta Lactamase	protection	GGTTACGGACAAGGTGAAATACTGAT	TGTCTTTTAATAAGTGAGGTGCGTTAATA
NDM1	ARG	Beta Lactamase	deactivate	ATTAGCCGCTGCATTGAT	CATGTCGAGATAGGAAGTG
pbp	ARG	Beta Lactamase	protection	CCGGTGCCATTGGTTTAGA	AAAATAGCCGCCCCAAGATT
pbp2x	ARG	Beta Lactamase	protection	TTTCATAAGTATCTGGACATGGAAGAA	CCAAAGGAAACTTGCTTGAGATTAG

Pbp5	ARG	Beta Lactamase	protection	GGCGAACTTCTAATTAATCCTATCCA	CGCCGATGACATTCTTCTTATCTT
penA	ARG	Beta Lactamase	protection	AGACGGTAACGTATAACTTTTTGAAAGA	GCGTGTAGCCGGCAATG
carB	ARG	MLSB	efflux	GGAGTGAGGCTGACCGTAGAAG	ATCGGCGAAACGCACAAA
ereA	ARG	MLSB	deactivate	CCTGTGGTACGGAGAATTCATGT	ACCGCATTCGCTTTGCTT
ereB	ARG	MLSB	deactivate	GCTTTATTTCAGGAGGCGGAAT	TTTTAAATGCCACAGCACAGAATC
erm(34)	ARG	MLSB	protection	GCGCGTTGACGACGATTT	TGGTCATACTCGACGGCTAGAAC
erm(35)	ARG	MLSB	protection	TTGAAAACGATGTTGCATTAAGTCA	TCTATAATCACAACTAACCACTTGAACGT
erm(36)	ARG	MLSB	protection	GGCGGACCGACTTGCAT	TCTGCGTTGACGACGGTTAC
ermA	ARG	MLSB	protection	TTGAGAAGGGATTTGCGAAAAG	ATATCCATCTCCACCATTAATAGTAAACC
ermA/ermTR	ARG	MLSB	protection	ACATTTTACCAAGGAACTTGTGGAA	GTGGCATGACATAAACCTTCATCA
ermB	ARG	MLSB	protection	TAAAGGGCATTTAACGACGAAACT	TTTATACCTCTGTTTGTTAGGGAATTGAA
ermC	ARG	MLSB	protection	TTTGAAATCGGCTCAGGAAAA	ATGGTCTATTTCAATGGCAGTTACG
ermF	ARG	MLSB	protection	CAGCTTTGGTTGAACATTTACGAA	AAATTCCTAAAATCACAACCGACAA
ermJ/ermD	ARG	MLSB	protection	GGACTCGGCAATGGTCAGAA	CCCCGAAACGCAATATAATGTT
ermK-01	ARG	MLSB	protection	GTTTGATATTGGCATTGTCAGAGAAA	ACCATTGCCGAGTCCACTTT
ermK-02	ARG	MLSB	protection	GAGCCGCAAGCCCCTTT	GTGTTTCATTTGACGCGGAGTAA
ermT-01	ARG	MLSB	protection	GTTCACTAGCACTATTTTTAATGACAGAAGT	GAAGGGTGTCTTTTTAATACAATTAACGA
ermT-02	ARG	MLSB	protection	GTAAAATCCCTAGAGAATACTTTCATCCA	TGAGTGATATTTTTGAAGGGTGTCTT
ermX	ARG	MLSB	protection	GCTCAGTGGTCCCCATGGT	ATCCCCCCGTCAACGTTT
ermY	ARG	MLSB	protection	TTGTCTTTGAAAGTGAAGCAACAGT	TAACGCTAGAGAACGATTTGTATTGAG
lmrA-01	ARG	MLSB	efflux	TCGACGTGACCGTAGTGAACA	CGTGACTACCCAGGTGAGTTGA
lnuA-01	ARG	MLSB	deactivate	TGACGCTCAACACACTCAAAAA	TTCATGCTTAAGTTCCATACGTGAA
lnuB-01	ARG	MLSB	deactivate	TGAACATAATCCCCTCGTTTAAAGAT	TAATTGCCCTGTTTCATCGTAAATAA
lnuB-02	ARG	MLSB	deactivate	AAAGGAGAAGGTGACCAATACTCTGA	GGAGCTACGTCAAACAACCAGTT
lnuC	ARG	MLSB	deactivate	TGGTCAATATAACAGATGTAAACCAGATTT	CACCCCAGCCACCATCAA
matA/mel	ARG	MLSB	efflux	TAGTAGGCAAGCTCGGTGTTGA	CCTGTGCTATTTTAAGCCTTGTTTCT
mdtA	ARG	MLSB	efflux	CCTAACGGGCGTGACTTCA	TTCACCTGTTTCAAGGGTCAAA
mefA	ARG	MLSB	efflux	CCGTAGCATTGGAACAGCTTTT	AAACGGAGTATAAGAGTGCTGCAA
mphA-01	ARG	MLSB	deactivate	CTGACGCGCTCCGTGTT	GGTGGTGCATGGCGATCT
mphA-02	ARG	MLSB	deactivate	TGATGACCCTGCCATCGA	TTCGCGAGCCCCTCTTC
mphB	ARG	MLSB	deactivate	CGCAGCGCTTGATCTTGTAG	TTACTGCATCCATACGCTGCTT
mphC	ARG	MLSB	deactivate	CGTTTGAAGTACCGAATTGGAAA	GCTGCGGGTTTGCCTGTA
msrA-01	ARG	MLSB	efflux	CTGCTAACACAAGTACGATTCCAAAT	TCAAGTAAAGTTGTCTTACCTACACCATT
msrC-01	ARG	MLSB	efflux	TCAGACCGGATCGGTTGTC	CCTATTTTTTGGAGTCTTCTCTCTAATGTT
oleC	ARG	MLSB	efflux	CCCGGAGTCGATGTTCGA	GCCGAAGACGTACACGAACAG
pikR1	ARG	MLSB	protection	TCGACATGCGTGACGAGATT	CCGCGAATTAGGCCAGAA
pikR2	ARG	MLSB	protection	TCGTGGGCCAGGTGAAGA	TTCCCCTTGCCGGTGAA

vatB-01	ARG	MLSB	deactivate	GGAAAAAGCAACTCCATCTCTTGA	TCCTGGCATAACAGTAACATTCTGA
vatB-02	ARG	MLSB	deactivate	TTGGGAAAAAGCAACTCCATCT	CAATCCACACATCATTTCCAACA
vatC-01	ARG	MLSB	deactivate	CGGAAATTGGGAACGATGTT	GCAATAATAGCCCCGTTTCCTA
vatC-02	ARG	MLSB	deactivate	CGATGTTTGGATTGGACGAGAT	GCTGCAATAATAGCCCCGTTT
vatE-01	ARG	MLSB	deactivate	GGTGCCATTATCGGAGCAAAT	TTGGATTGCCACCGACAAT
vatE-02	ARG	MLSB	deactivate	GACCGTCCTACCAGGCGTAA	TTGGATTGCCACCGACAATT
vgaA-01	ARG	MLSB	efflux	CGAGTATTGTGGAAAGCAGCTAGTT	CCCGTACCGTTAGAGCCGATA
vgaA-02	ARG	MLSB	efflux	GACGGGTATTGTGGAAAGCAA	TTTCCTGTACCATTAGATCCGATAATT
vgb-01	ARG	MLSB	deactivate	AGGGAGGGTATCCATGCAGAT	ACCAAATGCGCCCGTTT
vgbB-01	ARG	MLSB	efflux	CAGCCGGATTCTGGTCCTT	TACGATCTCCATTCAATTGGGTAAA
vgbB-02	ARG	MLSB	deactivate	ATACGAGCTGCCTAATAAAGGATCTT	TGTGAACCACAGGGCATTATCA
sul2	ARG	Sulfonamide	protection	TCATCTGCCAAACTCGTCGTTA	GTCAAAGAACGCCGCAATGT
sul1	ARG	Sulfonamide	protection	CAGCGCTATGCGCTCAAG	ATCCCGCTGCGCTGAGT
sulA/folP-01	ARG	Sulfonamide	protection	CAGGCTCGTAAATTGATAGCAGAAG	CTTTCCTTGCGAATCGCTTT
sulA/folP-03	ARG	Sulfonamide	protection	CACGGCTTCGGCTCATGT	TGCCATCCTGTGACTAGCTACGT
dfrA1	ARG	Sulfonamide	deactivate	GGAATGGCCCTGATATTCCA	AGTCTTGCGTCCAACCAACAG
dfrA12	ARG	Sulfonamide	deactivate	CCTCTACCGAACCGTCACACA	GCGACAGCGTTGAAACAACTAC
folA	ARG	Sulfonamide	deactivate	CGAGCAGTTCCTGCCAAAG	CCCAGTCATCCGGTTCATAATC
tet(32)	ARG	Tetracycline	protection	CCATTACTTCGGACAACGGTAGA	CAATCTCTGTGAGGGCATTTAACA
tet(34)	ARG	Tetracycline	unknown	CTTAGCGCAAACAGCAATCAGT	CGGTGATACAGCGCGTAAACT
tet(35)	ARG	Tetracycline	unknown	ACCCCATGACGTACCTGTAGAGA	CAACCCACACTGGCTACCAGTT
tet(36)-01	ARG	Tetracycline	protection	AGAATACTCAGCAGAGGTCAGTTCCT	TGGTAGGTCGATAACCCGAAAAT
tet(36)-02	ARG	Tetracycline	protection	TGCAGGAAAGACCTCCATTACAG	CTTTGTCCACACTTCCACGTACTATG
tet(37)	ARG	Tetracycline	unknown	GAGAACGTTGAAAAGGTGGTGAA	AACCAAGCCTGGATCAGTCTCA
tetA-01	ARG	Tetracycline	efflux	GCTGTTTGTTCTGCCGGAAA	GGTTAAGTTCCTTGAACGCAAACT
tetA-02	ARG	Tetracycline	efflux	CTCACCAGCCTGACCTCGAT	CACGTTGTTATAGAAGCCGCATAG
tetB-01	ARG	Tetracycline	efflux	AGTGCGCTTTGGATGCTGTA	AGCCCCAGTAGCTCCTGTGA
tetB-02	ARG	Tetracycline	efflux	GCCCAGTGCTGTTGTTGTCAT	TGAAAGCAAACGGCCTAAATACA
tetC-01	ARG	Tetracycline	efflux	CATATCGCAATACATGCGAAAAA	AAAGCCGCGGTAAATAGCAA
tetC-02	ARG	Tetracycline	efflux	ACTGGTAAGGTAAACGCCATTGTC	ATGCATAAACCAGCCATTGAGTAAG
tetD-01	ARG	Tetracycline	efflux	TGCCGCGTTTGATTACACA	CACCAGTGATCCCGGAGATAA
tetD-02	ARG	Tetracycline	efflux	TGTCATCGCGCTGGTGATT	CATCCGCTTCCGGGAGAT
tetE	ARG	Tetracycline	efflux	TTGGCGCTGTATGCAATGAT	CGACGACCTATGCGATCTGA
tetG-01	ARG	Tetracycline	efflux	TCAACCATTGCCGATTCGA	TGGCCCGGCAATCATG
tetG-02	ARG	Tetracycline	efflux	CATCAGCGCCGGTCTTATG	CCCCATGTAGCCGAACCA
tetH	ARG	Tetracycline	efflux	TTTGGGTCATCTTACCAGCATTAA	TTGCGCATTATCATCGACAGA
tetJ	ARG	Tetracycline	efflux	GGGTGCCGCATTAGATTACCT	TCGTCCAATGTAGAGCATCCATA

tetK	ARG	Tetracycline	efflux	CAGCAGTCATTGGAAAATTATCTGATTATA	CCTTGTACTAACCTACCAAAAATCAAAATA
tetL-01	ARG	Tetracycline	efflux	AGCCCGATTTATTCAAGGAATTG	CAAATGCTTTCCCCCTGTTCT
tetL-02	ARG	Tetracycline	efflux	ATGGTTGTAGTTGCGCGCTATAT	ATCGCTGGACCGACTCCTT
tetM-01	ARG	Tetracycline	protection	CATCATAGACACGCCAGGACATAT	CGCCATCTTTTGCAGAAATCA
tetM-02	ARG	Tetracycline	protection	TAATATTGGAGTTTTAGCTCATGTTGATG	CCTCTCTGACGTTCTAAAAGCGTATTAT
tetO-01	ARG	Tetracycline	protection	ATGTGGATACTACAACGCATGAGATT	TGCCTCCACATGATATTTTTCCT
tetPA	ARG	Tetracycline	efflux	AGTTGCAGATGTGTATAGTCGTAAACTATCTATT	TGCTACAAGTACGAAAACAAAACTAGAA
tetPB-01	ARG	Tetracycline	protection	ACACCTGGACACGCTGATTTT	ACCGTCTAGAACGCGGAATG
tetPB-02	ARG	Tetracycline	protection	TGATACACCTGGACACGCTGAT	CGTCCAAAACGCGGAATG
tetPB-03	ARG	Tetracycline	protection	TGGGCGACAGTAGGCTTAGAA	TGACCCTACTGAAACATTAGAAATATACCT
tetPB-05	ARG	Tetracycline	protection	CTGAAGTGGAGCGATCATTCC	CCCTCAACGGCAGAAATAACTAA
tetQ	ARG	Tetracycline	protection	CGCCTCAGAAGTAAGTTCATACACTAAG	TCGTTCATGCGGATATTATCAGAAT
tetR-02	ARG	Tetracycline	efflux	CGCGATAGACGCCTTCGA	TCCTGACAACGAGCCTCCTT
tetR-03	ARG	Tetracycline	efflux	CGCGATGGAGCAAAAGTACAT	AGTGAAAAACCTTGTTGGCATAAAA
tetS	ARG	Tetracycline	protection	TTAAGGACAAACTTTCTGACGACATC	TGTCTCCCATTGTTCTGGTTCA
tetT	ARG	Tetracycline	protection	CCATATAGAGGTTCCACCAAATCC	TGACCCTATTGGTAGTGGTTCTATTG
tetU-01	ARG	Tetracycline	unknown	GTGGCAAAGCAACGGATTG	TGCGGGCTTGCAAAACTATC
tetV	ARG	Tetracycline	efflux	GCGGGAACGACGATGTATATC	CCGCTATCTCACGACCATGAT
tetW-01	ARG	Tetracycline	protection	ATGAACATTCCCACCGTTATCTTT	ATATCGGCGGAGAGCTTATCC
tetX	ARG	Tetracycline	unknown	AAATTTGTTACCGACACGGAAGTT	CATAGCTGAAAAAATCCAGGACAGTT
vanA	ARG	Vancomycin	protection	AAAAGGCTCTGAAAACGCAGTTAT	CGGCCGTTATCTTGTAAAAACAT
vanB-01	ARG	Vancomycin	protection	TTGTCGGCGAAGTGGATCA	AGCCTTTTTCCGGCTCGTT
vanC-01	ARG	Vancomycin	protection	ACAGGGATTGGCTATGAACCAT	TGACTGGCGATGATTTGACTATG
vanC-02	ARG	Vancomycin	protection	CCTGCCACAATCGATCGTT	CGGCTTCATTCGGCTTGATA
vanC-03	ARG	Vancomycin	protection	AAATCAATACTATGCCGGGCTTT	CCGACCGCTGCCATCA
vanC1	ARG	Vancomycin	protection	AGGCGATAGCGGGTATTGAA	CAATCGTCAATTGCTCATTTCC
vanC2/vanC3	ARG	Vancomycin	protection	TTTGACTGTCGGTGCTTGTGA	TCAATCGTTTCAGGCAATGG
vanG	ARG	Vancomycin	protection	ATTTGAATTGGCAGGTATACAGGTTA	TGATTTGTCTTTGTCCATACATAATGC
vanHB	ARG	Vancomycin	protection	GAGGTTTCCGAGGCGACAA	CTCTCGGCGGCAGTCGTAT
vanHD	ARG	Vancomycin	protection	GTGGCCGATTATACCGTCATG	CGCAGGTCATTCAGGCAAT
vanRA-01	ARG	Vancomycin	protection	CCCTTACTCCCACCGAGTTTT	TTCGTCGCCCCATATCTCAT
vanRA-02	ARG	Vancomycin	protection	CCACTCCGGCCTTGTCATT	GCTAACCACATTCCCCTTGTTTT
vanRB	ARG	Vancomycin	protection	GCCCTGTCGGATGACGAA	TTACATAGTCGTCTGCCTCTGCAT
vanRC	ARG	Vancomycin	protection	TGCGGGAAAAACTGAACGA	CCCCCCATACGGTTTTGATTA
vanRC4	ARG	Vancomycin	protection	AGTGCTTTGGCTTATCTCGAAAA	TCCGGCAGCATCACATCTAA
vanRD	ARG	Vancomycin	protection	TTATAATGGCAAGGATGCACTAAAGT	CGTCTACATCCGGAAGCATGA
vanSA	ARG	Vancomycin	protection	CGCGTCATGCTTTCAAAATTC	TCCGCAGAAAGCTCAATTTGTT

vanSB	ARG	Vancomycin	protection	GCGCGGCAAATGACAAC	TTTGCCATTTTATTCGCACTGT
vanSC-01	ARG	Vancomycin	protection	ATCAACTGCGGGAGAAAAGTCT	TCCGCTGTTCCGCTTCTT
vanSC-02	ARG	Vancomycin	protection	GCCATCAGCGAGTCTGATGA	CAGCTGGGATCGTTTTTCCTT
vanTC-01	ARG	Vancomycin	protection	CACACGCATTTTTTCCCATCTAG	CAGCCAACAGATCATCAAAACAA
vanTC-02	ARG	Vancomycin	protection	ACAGTTGCCGCTGGTGAAG	CGTGGCTGGTCGATCAAAA
vanTE	ARG	Vancomycin	protection	GTGGTGCCAAGGAAGTTGCT	CGTAGCCACCGCAAAAAAAT
vanTG	ARG	Vancomycin	protection	CGTGTAGCCGTTCCGTTCTT	CGGCATTACAGGTATATCTGGAAA
vanWB	ARG	Vancomycin	protection	CGGACAAAGATACCCCCTATAAAG	AAATAGTAAATTGCTCATCTGGCACAT
vanWG	ARG	Vancomycin	protection	ACATTTTCATTTTGGCAGCTTGTAC	CCGCCATAAGAGCCTACAATCT
vanXA	ARG	Vancomycin	protection	CGCTAAATATGCCACTTGGGATA	TCAAAAGCGATTCAGCCAACT
vanXB	ARG	Vancomycin	protection	AGGCACAAAATCGAAGATGCTT	GGGTATGGCTCATCAATCAACTT
vanXD	ARG	Vancomycin	protection	TAAACCGTGTTATGGGAACGAA	GCGATAGCCGTCCCATAAGA
vanYB	ARG	Vancomycin	protection	GGCTAAAGCGGAAGCAGAAA	GATATCCACAGCAAGACCAAGCT
vanYD-01	ARG	Vancomycin	protection	AAGGCGATACCCTGACTGTCA	ATTGCCGGACGGAAGCA
vanYD-02	ARG	Vancomycin	protection	CAAACGGAAGAGAGGTCACTTACA	CGGACGGTAATAGGGACTGTTC
acrA-01	ARG	Multidrug	efflux	CAACGATCGGACGGGTTTC	TGGCGATGCCACCGTACT
acrA-02	ARG	Multidrug	efflux	GGTCTATCACCCTACGCGCTATC	GCGCGCACGAACATACC
acrA-03	ARG	Multidrug	efflux	CAGACCCGCATCGCATATT	CGACAATTTCGCGCTCATG
acrA-04	ARG	Multidrug	efflux	TACTTTGCGCGCCATCTTC	CGTGCGCGAACGAACAT
acrB-01	ARG	Multidrug	efflux	AGTCGGTGTTCGCCGTTAAC	CAAGGAAACGAACGCAATACC
acrR-01	ARG	Multidrug	efflux	GCGCTGGAGACACGACAAC	GCCTTGCTGCGAGAACAAA
acrR-02	ARG	Multidrug	efflux	GATGATACCCCCTGCTGTGAGA	ACCAAACAAGAAGCGCAAGAA
adeA	ARG	Multidrug	efflux	CAGTTCGAGCGCCTATTTCTG	CGCCCTGACCGACCAAT
acrA-05	ARG	Multidrug	efflux	CGTGCGCGAACGAACA	ACTTTGCGCGCCATCTTC
acrF	ARG	Multidrug	efflux	GCGGCCAGGCACAAAA	TACGCTCTTCCCACGGTTTC
ceoA	ARG	Multidrug	efflux	ATCAACACGGACCAGGACAAG	GGAAAGTCCGCTCACGATGA
cmeA	ARG	Multidrug	efflux	GCAGCAAAGAAGAAGCACCAA	AGCAGGGTAAGTAAAACTAAGTGGTAAATCT
cmr	ARG	Multidrug	efflux	CGGCATCGTCAGTGGAATT	CGGTTCCGAAAAAGATGGAA
emrD	ARG	Multidrug	efflux	CTCAGCAGTATGGTGGTAAGCATT	ACCAGGCGCCGAAGAAC
marR-01	ARG	Multidrug	efflux	GCGGCGTACTGGTGAAGCTA	TGCCCTGGTCGTTGATGA
mdetl1	ARG	Multidrug	efflux	ATACAGCAGTGGATATTGGTTTAATTGT	TGCATAAGGTGAATGTTCCATGA
mdtE/yhiU	ARG	Multidrug	efflux	CGTCGGCGCACTCGTT	TCCAGACGTTGTACGGTAACCA
mepA	ARG	Multidrug	efflux	ATCGGTCGCTCTTCGTTCAC	ATAAATAGGATCGAGCTGCTGGAT
mexA	ARG	Multidrug	efflux	AGGACAACGCTATGCAACGAA	CCGGAAAGGGCCGAAAT
mexD	ARG	Multidrug	efflux	TTGCCACTGGCTTTCATGAG	CACTGCGGAGAACTGTCTGTAGA
mexE	ARG	Multidrug	efflux	GGTCAGCACCGACAAGGTCTAC	AGCTCGACGTACTTGAGGAACAC

mexF	ARG	Multidrug	efflux	CCGCGAGAAGGCCAAGA	TTGAGTTCGGCGGTGATGA
mtrC-01	ARG	Multidrug	efflux	GGACGGGAAGATGGTCCAA	CGTAGCGTTCCGGTTCGAT
mtrC-02	ARG	Multidrug	efflux	CGGAGTCCATCGACCATTTG	ATCGTCGGCAAGGAGAATCA
mtrD-02	ARG	Multidrug	efflux	GGTCGGCACGCTCTTGTC	TGAAGAATTTGCGCACCACTAC
mtrD-03	ARG	Multidrug	efflux	CCGCCAAGCCGATATAGACA	GGCCGGGTTGCCAAA
oprD	ARG	Multidrug	efflux	ATGAAGTGGAGCGCCATTG	GGCCACGGCGAACTGA
oprJ	ARG	Multidrug	efflux	ACGAGAGTGGCGTCGACAA	AAGGCGATCTCGTTGAGGAA
pmrA	ARG	Multidrug	efflux	TTTGCAGGTTTTGTTCCTAATGC	GCAGAGCCTGATTTCTCCTTTG
putative multidrug	ARG	Multidrug	efflux	AATTTTGCCGATTATTGCTGAAA	GATTGTCATCATTCGTTTATCACCAA
qac	ARG	Multidrug	efflux	CAATAATAACCGAAATAATAGGGACAAGTT	AATAAGTGTTCCTAGTGTTGGCCATAG
qacA	ARG	Multidrug	efflux	TGGCAATAGGAGCTATGGTGTTT	AAGGTAACACTATTTTCGGTCCAAATC
qacA/qacB	ARG	Multidrug	efflux	TTTAGGCAGCCTCGCTTCA	CCGAATCCAAATAAAACCCAATAA
qacEdelta1-01	ARG	Multidrug	efflux	TCGCAACATCCGCATTAAAA	ATGGATTTCAGAACCAGAGAAAGAAA
qacEdelta1-02	ARG	Multidrug	efflux	CCCCTTCCGCCGTTGT	CGACCAGACTGCATAAGCAACA
qacH-01	ARG	Multidrug	efflux	GTGGCAGCTATCGCTTGGAT	CCAACGAACGCCCACAA
qacH-02	ARG	Multidrug	efflux	CATCGTGCTTGTGGCAGCTA	TGAACGCCCAGAAGTCTAGTTTT
rarD-02	ARG	Multidrug	efflux	TGACGCATCGCGTGATCT	AAATTTTCTGTGGCGTCTGAATC
sdeB	ARG	Multidrug	efflux	CACTACCGCTTCCGCACTTAA	TGAAAAAACGGGAAAAGTCCAT
tolC-01	ARG	Multidrug	efflux	GGCCGAGAACCTGATGCA	AGACTTACGCAATTCCGGGTTA
tolC-02	ARG	Multidrug	efflux	CAGGCAGAGAACCTGATGCA	CGCAATTCCGGGTTGCT
tolC-03	ARG	Multidrug	efflux	GCCAGGCAGAGAACCTGATG	CGCAATTCCGGGTTGCT
ttgA	ARG	Multidrug	efflux	ACGCCAATGCCAAACGATT	GTCACGGCGCAGCTTGA
ttgB	ARG	Multidrug	efflux	TCGCCCTGGATGTACACCTT	ACCATTGCCGACATCAACAAC
yceE/mdtG-01	ARG	Multidrug	efflux	TGGCACAAAATATCTGGCAGTT	TTGTGTGGCGATAAGAGCATTAG
yceE/mdtG-02	ARG	Multidrug	efflux	TTATCTGTTTTCTGCTCACCTTCTTTT	GCGTGGTGACAAACAGGCTTA
yceL/mdtH-01	ARG	Multidrug	efflux	TCGGGATGGTGGGCAAT	CGATAACCGAGCCGATGTAGA
yceL/mdtH-02	ARG	Multidrug	efflux	CGCGTGAAACCTTAAGTGCTT	AGACGGCTAAACCCCATATAGCT
yceL/mdtH-03	ARG	Multidrug	efflux	CTGCCGTTAAATGGATGTATGC	ACTCCAGCGGGCGATAGG
yidY/mdtL-01	ARG	Multidrug	efflux	GCAGTTGCATATCGCCTTCTC	CTTCCCGGCAAACAGCAT
yidY/mdtL-02	ARG	Multidrug	efflux	TGCTGATCGGGATTCTGATTG	CAGGCGCGACGAACATAAT
fabK	ARG	other	deactivate	TTTCAGCTCAGCACTTTGGTCAT	AAGGCATCTTTTTCAGCCAGTTC
imiR	ARG	other	unknown	CCGGACTAGAGCTTCATGTAAGC	CCCACGCGGTACTCTTGTAAA
nisB	ARG	other	unknown	GGGAGAGTTGCCGATGTTGTA	AGCCACTCGTTAAAGGGCAAT
speA	ARG	other	unknown	GCAAGAGGTATTTGCTCAACAAGA	CAGGGTCACCCTCATAAAGAAAA
bacA-01	ARG	other	deactivate	CGGCTTCGTGACCTCGTT	ACAATGCGATACCAGGCAAAT
bacA-02	ARG	other	deactivate	TTCCACGACACGATTAAGTCATTG	CGGCTCTTTCGGCTTCAG

fosB	ARG	other	deactivate	TCACTGTAACTAATGAAGCATTAGACCAT	CCATCTGGATCTGTAAAGTAAAGAGATC
fosX	ARG	other	deactivate	GATTAAGCCATATCACTTTAATTGTGAAAG	TCTCCTTCCATAATGCAAATCCA
nimE	ARG	other	unknown	TGCGCCAAGATAGGGCATA	GTCGTGAATTCGGCAGGTTTA
pncA	ARG	other	unknown	GCAATCGAGGCGGTGTTC	TTGCCGCAGCCAATTCA
sat4	ARG	other	deactivate	GAATGGGCAAAGCATAAAAACTTG	CCGATTTTGAAACCACAATTATGATA

Supplementary Table 2. Biomes included in this study. The biome classification was done based on vegetation field information and climatic information from the Köppen classification[16].

Ecosystem	n	Details
Boreal Forests	64	Northern Hemisphere, high latitude Forests (>50º)
Cold Forests	87	Continental Forests excluding boreal Forests
Temp. Forests	260	Temperate Forests<br>(67% Subtropical, 19% Oceanic and 14% Mediterranean)
Trop. Forests	24	Tropical Forests
Dry Forests	105	Arid Forests
Cold Grasslands	19	Continental Grasslands
Temp./trop. Grasslands	28	Temperate and tropical Grasslands
Dry Grasslands	47	Arid Grasslands
Cold Shrublands	3	Continental Shrublands
Temp./trop. Shrublands	30	Temperate and tropical Shrublands
Dry Shrublands	122	Arid Shrublands
Tundra	13	Polar climate
Croplands	210	Croplands including maize, soybean, rice and peanuts

Supplementary Table 3. Environmental factors included in our structural equation model.

Variables	Label	Variable	Units
ARGs	ARGs	Richness or proportion	Number of ARGs / Standardized relative abundance of ARGs
Spatial	Elevation	Elevation	m
	Spatial dissimilarity	(Euclidean distance) based on latitude and longitude (sine and cosine)	Decimal degrees
Climate	MAT	Mean annual temperature	ºC
	MAP	Mean annual precipitation	mm
	TSEA	Temperature Seasonality (standard deviation *100)	Unitless
	PSEA	Precipitation Seasonality (Coefficient of Variation)	%
Vegetation	Forests (F)	Location supporting Forests ecosystems	0/1
	Grasslands (G)	Location supporting Grasslands ecosystems	0/1
	Shrublands (S)	Location supporting Shrublands ecosystems	0/1
	Plant cover	Plant cover (vegetation cover)	% * 100
Soil	Fine texture (Clay+silt)	Fine texture	%
	pH	pH	Unitless
	Soil C	Soil carbon	%
	Soil C:N	Soil carbon to nitrogen ratio	Unitless
MGEs	MGEs	Richness and proportion	Number of MGEs / Standardized MGE relative abundance

Supplementary Table 4. Standardized direct effects of SEM on the proportion of soil ARGs.

Parameters			Direct effect	Bootstrap-P
PSEA	←	Spatial dissimilarity	-0.176	0.001
MAT	←	Spatial dissimilarity	-0.028	0.264
MAP	←	Spatial dissimilarity	-0.322	0.001
TSEA	←	Spatial dissimilarity	-0.292	0.001
PSEA	←	Elevation	0.218	0.001
MAT	←	Elevation	-0.200	0.001
MAP	←	Elevation	-0.199	0.001
TSEA	←	Elevation	-0.136	0.001
Forests	←	PSEA	0.114	0.001
Forests	←	MAT	-0.088	0.271
Forests	←	MAP	0.343	0.001
Forests	←	TSEA	0.029	0.715
Plant cover	←	PSEA	0.114	0.001
Plant cover	←	MAT	-0.502	0.002
Plant cover	←	MAP	0.677	0.001
Plant cover	←	TSEA	-0.513	0.002
Grasslands	←	PSEA	-0.072	0.090
Shrublands	←	PSEA	-0.052	0.190
Grasslands	←	MAT	-0.166	0.026
Shrublands	←	MAT	0.110	0.109
Grasslands	←	MAP	-0.231	0.001
Shrublands	←	MAP	-0.509	0.001
Grasslands	←	TSEA	-0.274	0.001
Shrublands	←	TSEA	-0.144	0.101
Plant cover	←	Spatial dissimilarity	-0.261	0.001
Forests	←	Spatial dissimilarity	0.245	0.002
Shrublands	←	Spatial dissimilarity	-0.154	0.005
Grasslands	←	Spatial dissimilarity	0.021	0.680
Plant cover	←	Elevation	-0.321	0.001
Forests	←	Elevation	0.015	0.733
Shrublands	←	Elevation	0.120	0.012
Grasslands	←	Elevation	0.105	0.051
Soil C:N	←	Forests	0.503	0.002
Soil C	←	Forests	0.342	0.001
pH	←	Forests	-0.418	0.002
pH	←	Plant cover	0.041	0.423
Soil C	←	Plant cover	0.008	0.895

Soil C:N	←	Plant cover	-0.037	0.527
pH	←	PSEA	-0.038	0.251
Soil C	←	PSEA	0.016	0.511
Soil C:N	←	PSEA	-0.099	0.009
pH	←	MAT	0.227	0.001
Soil C	←	MAT	-0.803	0.002
pH	←	MAP	-0.667	0.001
Soil C	←	MAP	0.013	0.747
Soil C:N	←	MAP	0.141	0.025
pH	←	TSEA	-0.034	0.568
Soil C	←	TSEA	-0.513	0.001
Soil C:N	←	TSEA	-0.314	0.001
Soil C:N	←	Shrublands	0.272	0.002
Soil C	←	Shrublands	0.002	0.936
pH	←	Shrublands	-0.111	0.002
Soil C:N	←	Grasslands	0.135	0.010
Soil C	←	Grasslands	-0.005	0.900
pH	←	Grasslands	-0.174	0.002
Soil C	←	Spatial dissimilarity	-0.251	0.001
Soil C:N	←	Spatial dissimilarity	0.004	0.937
pH	←	Spatial dissimilarity	-0.077	0.023
Fine texture	←	Plant cover	0.049	0.298
Fine texture	←	Forests	-0.376	0.001
Fine texture	←	Shrublands	-0.377	0.001
Fine texture	←	Grasslands	-0.331	0.001
Fine texture	←	PSEA	0.082	0.006
Fine texture	←	MAP	0.052	0.238
Fine texture	←	TSEA	0.249	0.002
Fine texture	←	Spatial dissimilarity	-0.143	0.001
Soil C:N	←	MAT	-0.513	0.001
Fine texture	←	Elevation	-0.114	0.004
pH	←	Elevation	0.153	0.001
Soil C	←	Elevation	-0.090	0.148
Soil C:N	←	Elevation	-0.074	0.098
Fine texture	←	MAT	-0.150	0.031
MGE proportion	←	Plant cover	0.052	0.420
MGE proportion	←	Forests	0.033	0.597
MGE proportion	←	Shrublands	0.144	0.006
MGE proportion	←	Grasslands	-0.007	0.796
MGE proportion	←	Soil C:N	0.079	0.139
MGE proportion	←	Soil C	0.007	0.835

MGE proportion	←	pH	0.022	0.615
MGE proportion	←	Spatial dissimilarity	-0.045	0.408
MGE proportion	←	PSEA	0.060	0.434
MGE proportion	←	MAT	-0.220	0.143
MGE proportion	←	MAP	0.077	0.327
MGE proportion	←	TSEA	-0.125	0.490
MGE richness	←	Soil C	-0.011	0.777
MGE richness	←	Soil C:N	0.032	0.626
MGE richness	←	pH	0.130	0.003
MGE richness	←	TSEA	0.199	0.018
MGE richness	←	MAP	0.008	0.933
MGE richness	←	MAT	-0.053	0.495
MGE richness	←	PSEA	0.133	0.002
MGE richness	←	Spatial dissimilarity	-0.008	0.822
MGE richness	←	Grasslands	-0.024	0.627
MGE richness	←	Shrublands	-0.055	0.272
MGE richness	←	Forests	-0.017	0.875
MGE richness	←	Plant cover	0.255	0.001
MGE richness	←	Fine texture	-0.067	0.083
MGE proportion	←	Fine texture	-0.152	0.009
MGE proportion	←	Elevation	-0.058	0.473
MGE richness	←	Elevation	0.063	0.213
ARG proportion	←	Plant cover	-0.053	0.169
ARG proportion	←	PSEA	0.111	0.001
ARG proportion	←	MAT	-0.245	0.003
ARG proportion	←	MAP	0.074	0.107
ARG proportion	←	TSEA	-0.183	0.032
ARG proportion	←	Forests	0.078	0.014
ARG proportion	←	Soil C:N	-0.039	0.130
ARG proportion	←	Soil C	0.003	0.916
ARG proportion	←	pH	0.007	0.777
ARG proportion	←	Shrublands	0.092	0.044
ARG proportion	←	Grasslands	0.023	0.430
ARG proportion	←	Spatial dissimilarity	-0.058	0.241
ARG proportion	←	MGE proportion	0.694	0.001
ARG proportion	←	MGE richness	-0.048	0.069
ARG proportion	←	Fine texture	-0.029	0.486
ARG proportion	←	Elevation	-0.085	0.050

Supplementary Table 5. Standardized direct effects of SEM on the richness of soil ARGs.

Parameters			Direct effect	Bootstrap-P
PSEA	←	Spatial dissimilarity	-0.176	0.001
MAT	←	Spatial dissimilarity	-0.028	0.264
MAP	←	Spatial dissimilarity	-0.322	0.001
TSEA	←	Spatial dissimilarity	-0.292	0.001
PSEA	←	Elevation	0.218	0.001
MAT	←	Elevation	-0.200	0.001
MAP	←	Elevation	-0.199	0.001
TSEA	←	Elevation	-0.136	0.001
Forests	←	PSEA	0.114	0.001
Forests	←	MAT	-0.088	0.271
Forests	←	MAP	0.343	0.001
Forests	←	TSEA	0.029	0.715
Plant cover	←	PSEA	0.114	0.001
Plant cover	←	MAT	-0.502	0.002
Plant cover	←	MAP	0.677	0.001
Plant cover	←	TSEA	-0.513	0.002
Grasslands	←	PSEA	-0.072	0.090
Shrublands	←	PSEA	-0.052	0.190
Grasslands	←	MAT	-0.166	0.026
Shrublands	←	MAT	0.110	0.109
Grasslands	←	MAP	-0.231	0.001
Shrublands	←	MAP	-0.509	0.001
Grasslands	←	TSEA	-0.274	0.001
Shrublands	←	TSEA	-0.144	0.101
Plant cover	←	Spatial dissimilarity	-0.261	0.001
Forests	←	Spatial dissimilarity	0.245	0.002
Shrublands	←	Spatial dissimilarity	-0.154	0.005
Grasslands	←	Spatial dissimilarity	0.021	0.680
Plant cover	←	Elevation	-0.321	0.001
Forests	←	Elevation	0.015	0.733
Shrublands	←	Elevation	0.120	0.012
Grasslands	←	Elevation	0.105	0.051
Soil C:N	←	Forests	0.503	0.002
Soil C	←	Forests	0.342	0.001
pH	←	Forests	-0.418	0.002
pH	←	Plant cover	0.041	0.423
Soil C	←	Plant cover	0.008	0.895

Soil C:N	←	Plant cover	-0.037	0.527
pH	←	PSEA	-0.038	0.251
Soil C	←	PSEA	0.016	0.511
Soil C:N	←	PSEA	-0.099	0.009
pH	←	MAT	0.227	0.001
Soil C	←	MAT	-0.803	0.002
pH	←	MAP	-0.667	0.001
Soil C	←	MAP	0.013	0.747
Soil C:N	←	MAP	0.141	0.025
pH	←	TSEA	-0.034	0.568
Soil C	←	TSEA	-0.513	0.001
Soil C:N	←	TSEA	-0.314	0.001
Soil C:N	←	Shrublands	0.272	0.002
Soil C	←	Shrublands	0.002	0.936
pH	←	Shrublands	-0.111	0.002
Soil C:N	←	Grasslands	0.135	0.010
Soil C	←	Grasslands	-0.005	0.900
pH	←	Grasslands	-0.174	0.002
Soil C	←	Spatial dissimilarity	-0.251	0.001
Soil C:N	←	Spatial dissimilarity	0.004	0.937
pH	←	Spatial dissimilarity	-0.077	0.023
Fine texture	←	Plant cover	0.049	0.298
Fine texture	←	Forests	-0.376	0.001
Fine texture	←	Shrublands	-0.377	0.001
Fine texture	←	Grasslands	-0.331	0.001
Fine texture	←	PSEA	0.082	0.006
Fine texture	←	MAP	0.052	0.238
Fine texture	←	TSEA	0.249	0.002
Fine texture	←	Spatial dissimilarity	-0.143	0.001
Soil C:N	←	MAT	-0.513	0.001
Fine texture	←	Elevation	-0.114	0.004
pH	←	Elevation	0.153	0.001
Soil C	←	Elevation	-0.090	0.148
Soil C:N	←	Elevation	-0.074	0.098
Fine texture	←	MAT	-0.150	0.031
MGE proportion	←	Plant cover	0.052	0.420
MGE proportion	←	Forests	0.033	0.597
MGE proportion	←	Shrublands	0.144	0.006
MGE proportion	←	Grasslands	-0.007	0.796
MGE proportion	←	Soil C:N	0.079	0.139
MGE proportion	←	Soil C	0.007	0.835

MGE proportion	←	pH	0.022	0.615
MGE proportion	←	Spatial dissimilarity	-0.045	0.408
MGE proportion	←	PSEA	0.060	0.434
MGE proportion	←	MAT	-0.220	0.143
MGE proportion	←	MAP	0.077	0.327
MGE proportion	←	TSEA	-0.125	0.490
MGE richness	←	Soil C	-0.011	0.777
MGE richness	←	Soil C:N	0.032	0.626
MGE richness	←	pH	0.130	0.003
MGE richness	←	TSEA	0.199	0.018
MGE richness	←	MAP	0.008	0.933
MGE richness	←	MAT	-0.053	0.495
MGE richness	←	PSEA	0.133	0.002
MGE richness	←	Spatial dissimilarity	-0.008	0.822
MGE richness	←	Grasslands	-0.024	0.627
MGE richness	←	Shrublands	-0.055	0.272
MGE richness	←	Forests	-0.017	0.875
MGE richness	←	Plant cover	0.255	0.001
MGE richness	←	Fine texture	-0.067	0.083
MGE proportion	←	Fine texture	-0.152	0.009
MGE proportion	←	Elevation	-0.058	0.473
MGE richness	←	Elevation	0.063	0.213
ARG richness	←	Plant cover	0.237	0.001
ARG richness	←	PSEA	0.054	0.065
ARG richness	←	MAT	0.106	0.107
ARG richness	←	MAP	-0.010	0.812
ARG richness	←	TSEA	0.309	0.001
ARG richness	←	Forests	0.335	0.001
ARG richness	←	Soil C:N	0.046	0.154
ARG richness	←	Soil C	-0.020	0.523
ARG richness	←	pH	-0.027	0.436
ARG richness	←	Shrublands	0.225	0.001
ARG richness	←	Grasslands	0.158	0.001
ARG richness	←	Spatial dissimilarity	-0.048	0.213
ARG richness	←	MGE proportion	0.003	0.907
ARG richness	←	MGE richness	0.476	0.001
ARG richness	←	Fine texture	-0.003	0.959
ARG richness	←	Elevation	0.048	0.179
